# Balsam Poplar Buds Extracts-Loaded Gels and Emulgels: Development, Biopharmaceutical Evaluation, and Biological Activity In Vitro

**DOI:** 10.3390/gels9100821

**Published:** 2023-10-17

**Authors:** Monika Jokubaite, Greta Pukenaite, Mindaugas Marksa, Kristina Ramanauskiene

**Affiliations:** 1 Department of Drug Chemistry, Faculty of Pharmacy, Lithuanian University of Health Sciences, Sukileliai Avenue 13, LT-50162 Kaunas, Lithuania; 2 Institute of Pharmaceutical Technologies, Faculty of Pharmacy, Lithuanian University of Health Sciences, Sukileliai Avenue 13, LT-50162 Kaunas, Lithuania; 3 Department of Clinical Pharmacy, Faculty of Pharmacy, Lithuanian University of Health Sciences, Sukileliai Avenue 13, LT-50162 Kaunas, Lithuania; pukenaite.g@gmail.com (G.P.); kristina.ramanauskiene@lsmu.lt (K.R.); 4 Department of Analytical & Toxicological Chemistry, Faculty of Pharmacy, Lithuanian University of Health Sciences, Sukileliai Avenue 13, LT-50162 Kaunas, Lithuania; mindaugas.marksa@lsmu.lt

**Keywords:** balsam poplar buds, gel, emulsion, emulgel

## Abstract

Balsam poplar buds have been used for wound healing and treating irritated skin in traditional medicine. Balsam poplar buds extracts exhibit anti-inflammatory, antioxidant, and antimicrobial effects. In recent years, scientific research has begun to validate some of these traditional uses, leading to an increased interest in balsam poplar buds as a potential source of natural remedies in modern medicine. The study aims to simulate semi-solid pharmaceutical forms with balsam poplar buds extract and evaluate their quality through biopharmaceutical research. The active compounds identified in Lithuanian poplar buds were *p*-coumaric acid, cinnamic acid, caffeic acid, galangin, pinocembrin, pinobanksin, and salicin. In gels, pH values ranged from 5.85 ± 0.05 to 5.95 ± 0.07. The determined pH values of emulgels ranged from 5.13 ± 0.05 to 5.66 ± 0.15. After 6 h, the release of active compounds from gels and emulgels ranged from 47.40 ± 2.41% to 71.17 ± 3.54. *p*-coumaric acid dominates in the balsam poplar buds extracts. The pH values of the prepared sem-solid pharmaceutical forms are suitable for use on the skin. The viscosity of the formulations depends on the amount of gelling agent. All formulations showed antioxidant activity. It is relevant to conduct a more extensive study on the influence of the chosen carrier on the release of active compounds from semi-solid formulations with an extract of balsam poplar buds.

## 1. Introduction

Natural origin products, such as plant extracts, essential oils, resins, and bee products, are frequently used to address skin problems [1]. Natural origin products may possess anti-inflammatory, antimicrobial, or regenerative properties [2,3]. Many plant species represent rich sources of phenolic compounds, encompassing phenolic acids, flavonoids, anthocyanins, tannins, and other bioactive compounds. Currently, there is increasing interest among researchers in the plant species of the *Salicaceae* family, known for their capacity to accumulate phenolic acids, flavonoids, and salicin derivatives [4,5]. Phenolic compounds encountered in the buds of poplar trees have been established to possess a spectrum of bioactive properties, including anti-inflammatory, antioxidant, antimicrobial, and wound-healing attributes [6,7,8,9,10]. These properties render them valuable assets in the therapeutic management of various disorders [11,12]. Studies conducted in Lithuania have unveiled a composition in balsam poplar buds extracts where the dominant compounds include *p*-coumaric acid, cinnamic acid, pinobanksin, galangin, and salicin [8]. Previous studies have found that aqueous extracts derived from poplar buds do not cause irritation when tested on the SIRC cell line, and this suggests that these extracts could be suitable for use in the development of eye drop formulations [9]. These buds extracts are also suitable for treating rheumatoid arthritis, as salicin, gallic acid, and salicortin found in poplar buds help to reduce joint pain and swelling [10,13,14]. Moreover, poplar buds possess antiseptic, diuretic, and anti-inflammatory properties [14,15]. UVB stimulates collagen degradation and inhibits pro-collagen biosynthesis, leading to collagen depletion and wrinkle formation, thereby promoting skin photoaging [16]. The negative effects of UVB exposure can be mitigated by using natural products with photoprotective properties. Scientific research has indicated that extracts obtained from balsam poplar buds collected in Lithuania exhibit protective properties against UV radiation, as well as antioxidant and antimicrobial effects [17]. Extracts from poplar buds can be used as active components in gels, creams, ointments, or emulsions aimed at treating inflammatory skin conditions or alleviating their symptoms, such as eczema, dermatitis, psoriasis, and related diseases [18]. For the treatment of skin diseases and alleviating their symptoms, the search for sources of natural active compounds is relevant. It is essential to explore new natural active compounds and semi-solid formulations that ensure formulation efficacy and are suitable for daily skin care and alleviating symptoms of inflammatory diseases. For the treatment of skin conditions, semi-solid pharmaceutical preparations such as ointments, creams, gels, and emulgels are commonly used [19,20]. A crucial aspect is selecting an appropriate base for the formulation. The chosen base must ensure the stability of the active substance, exhibit acceptable sensory properties, and act as a carrier for the active compound. Scientific studies have demonstrated that the release of active compounds from the base depends on its type and physicochemical properties [21,22]. A hydrophilic gel base provides sufficient hydration for various skin types, but if the skin is dry or affected by eczema, an emulsion system is preferable as a moisturizer [23]. Gels have limitations in delivering hydrophobic drugs, which can be overcome by using emulgels. An emulgel, as a topical drug delivery system, possesses a dual release control system—the gel and the emulsion phases [24,25,26]. During this investigation, gels, oil-in-water emulsions, and emulgels were selected for the modeling of formulations [27]. The chosen active substance is balsam poplar buds extract due to its existing antioxidant and antimicrobial properties. The aim of the study is to model semi-solid pharmaceutical forms with balsam poplar buds extract and evaluate their quality through biopharmaceutical investigations.

## 2. Results

### 2.1. Analysis of Active Compounds in Balsam Poplar Buds Extracts

**Figure 1:**
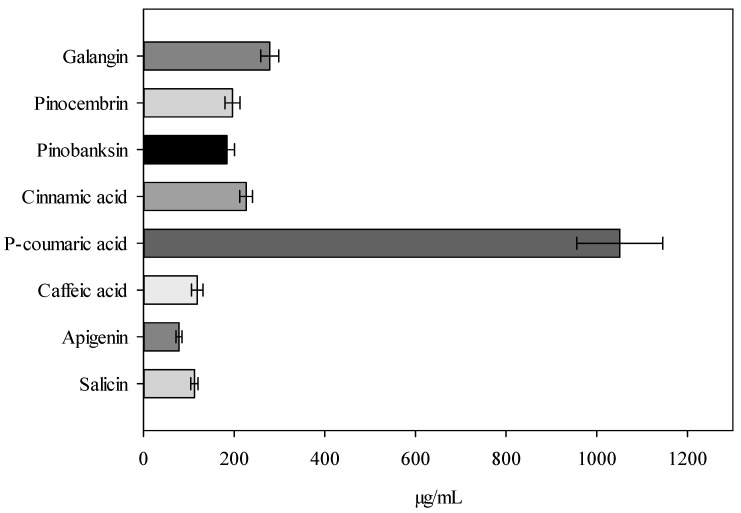
Content of phenolic compounds in balsam poplar buds extract (µg/mL, *n* = 3, SD—standard deviation).

Liquid ethanol extracts were prepared using the maceration method, macerating for 7 days at room temperature. The results presented in Figure 1 indicate that the dominant compound in the extracts is *p*-coumaric acid. These results confirm previous research findings describing *p*-coumaric acid as the dominant compound in balsam poplar buds extracts collected in Lithuania. Statistically significantly lower amounts of cinnamic acid and caffeic acid were detected compared to *p*-coumaric acid in the extracts (*p* < 0.05). The dominant flavonoids in the extract were determined as follows: galangin > pinocembrin > pinobanksin > apigenin. The total phenolic content in the ethanol extract was found to be 234.12 ± 22.03 mg GAE/g, and the total flavonoid content was 28.72 ± 4.74 mg RE/g.

### 2.2. Production of Gels with a Liquid Extract of Balsam Poplar Buds

**Figure 2:**
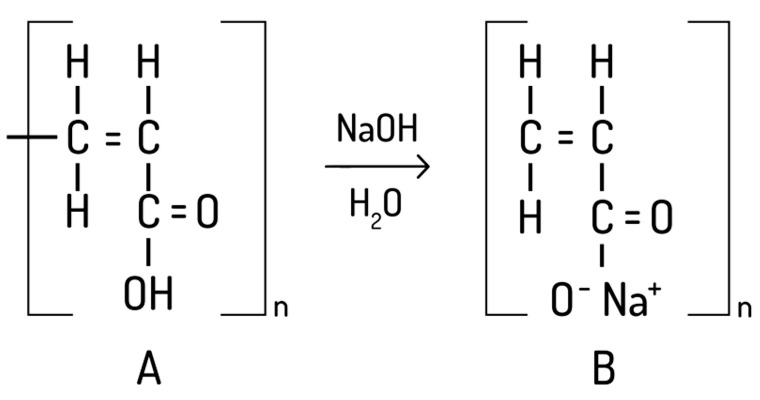
Chemical structure of polyacrylic acid A and sodium polyacrylate B.

Carbomers are acrylic acid polymers used in pharmaceutical processes for various purposes, such as suspending agents, gel bases, emulsifiers, binding agents, and artificial tears. Carbomer 980, a cross-linked polyacrylate polymer, offers a high viscosity and excellent thickening and suspending performance at low concentrations (Figure 2) [28,29]. During these studies, hydrogels with different concentrations of carbomer 980 were prepared. After preparing the gels, a 20% balsam poplar buds extract was incorporated (Table 1). Three different compositions of gels were produced, using different concentrations of carbomer: 0.5%, 1%, and 2%. The gels formulated with carbomer and balsam poplar buds extract exhibited a semi-solid consistency, were off-white in color, and had a pleasant aroma. In the subsequent phase of the research, an oil-in-water emulsion (E0) was prepared, with the composition selected based on an analysis of scientific literature. Table 1 presents the compositions of the emulsion (E0) and the emulsion containing the incorporated liquid poplar buds extract (E1). The prepared emulsion E0 had a semi-solid consistency, was white in color, and odorless. Emulsion E1, which contained the poplar buds extract, exhibited a semi-solid consistency, was lightly yellowish in color, and had a pleasant and specific aroma. The prepared emulsion system was homogeneous. The emulsion produced was suitable for application on the skin, with a proper pH value that did not cause skin irritation. The emulsion had a semi-solid consistency, which was confirmed by the consistency results. In the next stage, emulgels were formulated using carbomer gels and the oil-in-water emulsion (E0). The emulgels contained 20 g of liquid balsam poplar buds extract per 100 g of formulation. The compositions of the formulated emulgels are presented in Table 1. Three emulgel systems were formulated using carbomer as the base, and different concentrations of gels were used, which were prepared in the previous phase of the study. Additionally, all emulgels contained the oil-in-water emulsion. A 20% liquid poplar buds extract was incorporated into all emulgels. The formulated emulgels had a semi-solid consistency, were off-white in color, and had a pleasant aroma.

### 2.3. Investigation of the Physicochemical Properties of Semi-Solid Formulations with Balsam Poplar Buds Liquid Extract

In order to assess the stability of the formulations, it is important to determine their physicochemical properties. In this study, the pH of the gels was measured, and their viscosity was evaluated, with the values provided in Table 2.

From the results presented in Table 2, it is evident that the viscosity of the tested gels G1–G3 ranged from 225.3 ± 7.7 to 408.4 ± 14.3 mPa·s. The study results demonstrated that the viscosity of the prepared gels depends on the concentration of the gelling agent. No statistically significant difference (*p* > 0.05) in viscosity was observed when comparing carbomer gels with a gelling agent concentration of 0.5% and 1%. However, a statistically significant higher viscosity (*p* < 0.05) was found in gels containing 2% carbomer. The pH values of the gels were not statistically different (*p* > 0.05). The incorporation of balsam poplar buds liquid extract did not influence the pH values of the formulations compared to the gels without the active ingredient.

The prepared emulsion was suitable for skin application, exhibiting an appropriate pH value that did not cause skin irritation. The emulsion had a semi-solid consistency with a viscosity of 2641.0 ± 96.3 mPa·s. The pH values of all emulgels ranged from 5.87 ± 0.16 to 5.95 ± 0.22, with no statistically significant difference between the pH values (*p* > 0.05). From the data presented in Table 2, it can be seen that the viscosity of the emulgels is influenced by the selected gelling agent and its concentration. The highest viscosity was observed in Emulgel Eg3, which contains 2% carbomer. The lowest viscosity among the tested emulgels was found in Eg1, which contains the lowest amount of the gelling agent (0.5%).

### 2.4. In Vitro Release Test from Formulations

**Figure 3:**
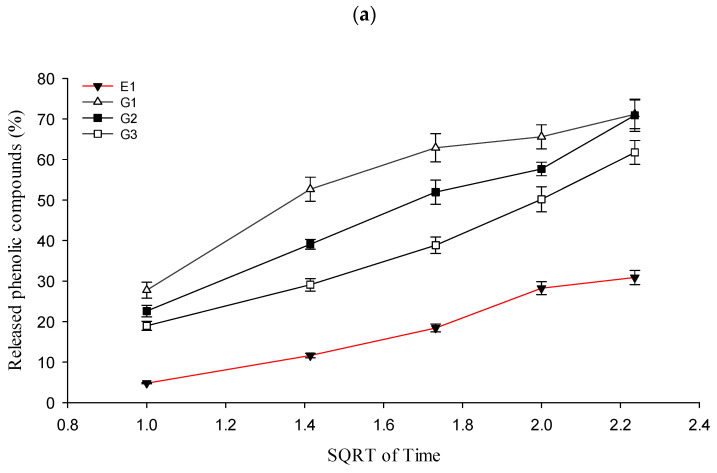

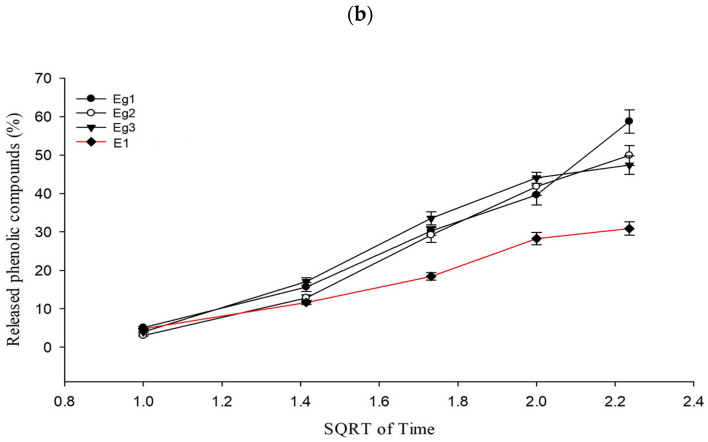
Percentage release of total phenolic compounds vs. square root of time (**a**) Percentage release of total phenolic compounds from Gels G1–G3 and Emulsion E1; (**b**) percentage release of total phenolic compounds from Emulgels Eg1–Eg3 and Emulsion E1 (*n* = 3, mean ± SD).

When assessing the quality of gels, it is essential to not only to evaluate the pH and viscosity values but also to conduct an in vitro drug release test. This study helps to assess the influence of selected gelling agents on the release of active components from the formulated gels. For the drug release test, 30% ethanol (*v*/*v*) was chosen as the acceptor medium. The percentage of released active compounds in semi-solid formulations with balsam poplar buds liquid extract is presented in Figure 3a,b.

From the data presented in Figure 3, it can be observed that the highest amount of active compounds was released from gels containing 0.5% carbomer, while the lowest amount was observed in gels containing 2% carbomer. Gel G2, with 1% carbomer, exhibited the highest release of active compounds. Gels G1 and G3 showed a lower release of phenolic compounds. Within the 1–4-h interval, all carbomer-based gels released between 18.96 ± 1.12% and 71.14 ± 3.54% of phenolic compounds. After 6 h of testing, the released amounts from Gels G1, G2, and G3 were 71.17 ± 3.54%, 70.90 ± 3.99%, and 63.74 ± 2.94%, respectively. Statistically significant differences (*p* < 0.05) were observed between the released amounts of active compounds from Gels G1 and G3, and G2 and G3. However, no statistically significant difference (*p* > 0.05) was found between the released amounts from Gels G1 and G2. Regarding the release of phenolic compounds from emulsion (E1), it steadily increased throughout the entire test period. Within the 1–4-h interval, the released amounts ranged from 4.82 ± 0.16% to 28.25 ± 1.59%. After 6 h, approximately 30.88 ± 1.74% of phenolic compounds were released from Emulsion E1. The released amounts of active compounds from Emulsion E1 were significantly lower (*p* < 0.05) compared to the carbomer-based gels G1–G3. In Figure 3b, the results of phenolic compounds released from Emulgels Eg1–Eg3 are presented. Within the 1–4-h interval, all emulgels based on carbomer released active compounds between 3.03 ± 0.19% and 44.08 ± 1.41%. After 6 h, the released amounts from Emulgels Eg1, Eg2, and Eg3 were 58.75 ± 3.01%, 49.91 ± 2.59%, and 47.40 ± 2.41%, respectively. A statistically significant difference (*p* < 0.05) was found between the released amounts of phenolic compounds from Emulgels Eg1 and Eg3 after 6 h. Additionally, a statistically significant difference (*p* < 0.05) was observed between the released amounts of phenolic compounds from Emulsion E1 and Emulgels Eg1–Eg3.

### 2.5. Evaluation of Semi-Solid Preparations’ Antioxidant Activity

**Figure 4:**
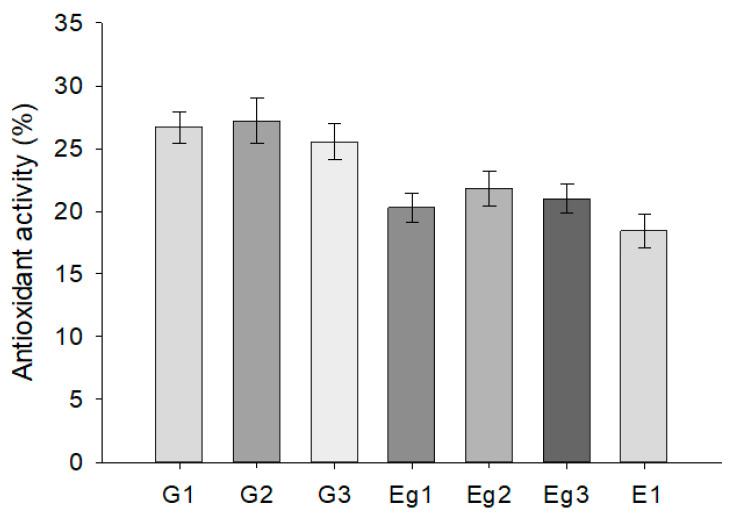
Antiradical activity in Gels G1–G3, Emulgels Eg1–Eg3, and Emulsion E1 in vitro release fractions after 6 h by the DPPH method (*n* = 3, mean ± SD).

In the final stage of the study, the antioxidant activity in vitro of the released phenolic compounds from semi-solid formulations with balsam poplar buds extract was evaluated after 6 h. The aim of this investigation was to examine whether the active compounds released from the formulated gels and emulgels exhibited antioxidant effects. The DPPH radical scavenging method was employed for the antioxidant evaluation. The results of the antioxidant assay are presented in Figure 4.

From the results presented in Figure 4, it can be observed that the antioxidant activity of Gels G1-G3 does not show statistically significant differences (*p* > 0.05). The concentration of the gelling agent does not influence the antiradical activity. The antioxidant activity of the emulsion is statistically significantly lower (*p* < 0.05) compared to Gels G1–G3. There is no statistically significant difference in the antioxidant activity among Emulgels Eg1–Eg3 (*p* > 0.05). Carbomer-based emulgels exhibit statistically significantly higher (*p* < 0.05) antioxidant activity compared to Emulgels Eg1–Eg3.

### 2.6. Antimicrobal Activity

After evaluating the antimicrobial activity of the ethanolic extract of balsam poplar buds and its dominant *p*-coumaric acid, a significantly stronger effect against strains of the Gram-positive bacteria *Staphylococcus aureus* and *Enterococcus faecalis* and strains of the fungus *Candida albicans* was determined, compared to strains of Gram-negative bacteria (Table 3). The antimicrobial effect against *Pseudomonas aeruginosa* bacterial strains has not been determined. Modeled semi-solid formulations did not exhibit antimicrobial activity against Gram-negative strains of the bacteria *Escherichia coli* and *Pseudomonas aeruginosa*. Gels and emulgels had an inhibitory zone greater than 5 mm against strains of *Staphylococcus aureus* bacteria and an inhibitory zone of less than 5 mm against strains of *Enterococcus faecalis* and *Candida albicals* microorganisms. No statistically significant difference in antimicrobial activity between gels from emulgels against *Staphylococcus aureus* bacterial strains was found (*p* > 0.05). Emulsion (E1) did not exhibit antimicrobial activity against *Candida albicans* fungal strains.

## 3. Discussion

In the scientific literature, it has been reported that balsam poplar buds collected in Lithuania are primarily composed of *p*-coumaric acid, with smaller quantities of caffeic acid, vanillin, chlorogenic, and ferulic acids, as well as trace amounts of flavonoids [8,9]. In this study, the extract also exhibited the dominance of *p*-coumaric acid, and the following flavonoids were identified and quantified: galangin, apigenin, pinocembrin, and pinobanksin. The identified active compounds in the poplar buds extract, based on data from the scientific literature, are known for their antioxidant and antibacterial effects [8,30,31,32]. Considering the research results, it is concluded that the produced poplar buds extract is suitable for incorporation into semi-solid pharmaceutical forms with protective properties due to its existing biological characteristics.

In order to model a hydrophilic base with semi-solid forms, a polymer called Carbopol was selected. Carbopol 980, a highly efficient synthetic polymer, is a suitable choice for semi-solid formulations due to its thickening and stabilizing properties. These properties can enhance product consistency, prolong drug release, and improve topical application, making it advantageous for both pharmaceutical and cosmetic applications [33]. The amount of gelling agent (Carbopol) had an impact on the viscosity of the gels, as an increase in the gelling agent resulted in a higher product viscosity. The balsam poplar buds extract was uniformly distributed within the Carbopol gels. The pH range of the gels formulated with the poplar buds extract using Carbopol as the base was found to be acceptable, falling within the recommended pH range for skin products (pH 5.5–7) [34]. Although gels are widely used in pharmaceutical formulations, they may not always be ideal for the user, considering the condition of the skin. Gels do not always ensure efficient transdermal delivery of the active ingredient and may not provide intense moisturization and nourishment for the skin [35,36]. Therefore, in further research, the liquid balsam poplar buds extract was incorporated into an emulsion system. Semi-solid emulsion systems are widely used in the production of skin products [37,38]. In this study, an oil-in-water (o/w) emulsion was chosen for formulation, with sunflower oil selected as the oily phase. Due to its high oleic acid content, sunflower oil is less prone to oxidative degradation, contains tocopherols that soften the skin, and is stable and safe [39,40]. Based on the scientific literature, Tween 60 and Span 60 were chosen as emulsifiers. The prepared emulsion system exhibited a suitable pH value. Comparing the viscosity of the produced emulsion with the viscosity of the gels, it can be observed that the emulsions have a higher viscosity than the tested gels. In order to create a more effective pharmaceutical form with the liquid balsam poplar buds extract, emulgels were selected. Emulgels are non-greasy, non-staining, skin-softening formulations that are easy to apply and remove, transparent, and have a longer shelf life. Emulgels are prepared by incorporating a gelled phase into an emulsion [41,42,43]. The viscosity of the emulgels depended on the concentration of the gelling agent: as the gelling agent concentration increased, so did the viscosity of the preparations. Among the emulgels based on Carbopol, the highest release of active compounds occurred when the gelling agent concentration and viscosity of the preparations were the lowest. Therefore, it can be concluded that the release is influenced by the gelling agent concentration and viscosity. Similar amounts of active compounds were released from both gels and emulgels, indicating that both formulations are suitable for delivering the balsam poplar buds extract. From the emulsion, the lowest amount of active compounds was released. The results of the release study confirm data from the scientific literature that the amount of released active compounds depends on the physicochemical properties of the chosen carrier. The antioxidant activity of the collected fractions after 6 h depends on the formulation’s ability to release the active compounds. Recently, many scientists have focused their attention on the research of plants, essential oils, and pure secondary metabolites as potential antimicrobial agents. Phytochemical antimicrobial substances can be categorized into several groups: phenols/polyphenols, terpenoids and essential oils, alkaloids, lectins, and polypeptides [44]. Our investigated ethanolic balsam poplar buds extracts exhibited significant antimicrobial activity against the Gram-positive bacteria *Staphylococcus aureus* and *Enterococcus faecalis* strains, as well as the fungal pathogen *Candida albicans*. The extracts and *p*-coumaric acid did not demonstrate significant antimicrobial activity against Gram-negative bacterial strains, such as *Pseudomonas aeruginosa*, and exhibited only weak inhibitory effects against *Escherichia coli* strains. Additionally, active compounds released from experimental semi-solid carriers in the study showed limited efficacy against Gram-positive bacterial strains and *Candida albicans* fungi. These results may indicate that the antimicrobial properties of the extract are selective, with varying levels of effectiveness against different bacterial species and fungal pathogens. Kis et al. reported that an extract from *Populus Nigra* L. exhibited inhibitory effects against Gram-positive bacteria, including *Streptococcus pyogenes*, *Streptococcus mutans*, and *Staphylococcus aureus* [31]. These findings align with our previous research results, which observed that balsam poplar buds exerted stronger antimicrobial effects on Gram-positive bacterial strains when compared to Gram-negative bacterial strains [8].

## 4. Conclusions

In the ethanolic extract of balsam poplar buds, the dominant compound is *p*-coumaric acid, along with other phenolic compounds. These components provide the potential for the extract to be used in local formulations for skin conditions. The chosen technology allowed the production of homogeneous, semi-solid preparations containing the liquid balsam poplar buds extract. The pH values of the produced semi-solid pharmaceutical forms with poplar buds extract confirm their suitability for topical application on the skin. The amount of released phenolic compounds from the prepared formulations depends on the selected base. The antioxidant activity of the collected acceptor-phase fractions depends on the amount of released active compounds. Active compounds released from experimental semi-solid formulations showed a limited efficacy against Gram-positive bacterial strains and fungi *Candida albicans*, such research results could be due to the low content of active compounds in the formulations. Balsam poplar buds extracts are a potential candidate of phenolic compounds with antimicrobial and antioxidant activity. The application of more efficient technologies for extracting active compounds in the production of poplar buds extracts can be one of the tools which can help to solve the problems of increasing the yield of active compounds. When developing semi-solid pharmaceutical forms with liquid balsam poplar buds extract, it is essential to conduct in vitro release studies through a semi-permeable membrane to select a suitable base as a carrier for the active substance.

## 5. Materials and Methods

### 5.1. Materials

Dried plant material of balsam poplar buds (“Jadvygos žolės”, Mažeikiai, Lithuania); 96% ethanol (AB “Vilniaus degtinė”, Lithuania); carbomer 980 (Lubrizol, OH, USA); hypromellose (Sigma-Aldrich, Saint Louis, MO, USA); sodium hydroxide, NaOH (Erba Lachema, Brno, Czech Republic); purified deionized water produced using the Milli-Q^®^ water purification system (Millipore, Burlington, MA, USA); sunflower oil (Sigma-Aldrich, Steinheim, Germany); Tween 60 (Sigma-Aldrich, Saint Louis, MO, USA); Span 60 (Sigma-Aldrich, Saint Louis, MO, USA); Folin–Ciocalteu reagent (Sigma-Aldrich, Saint Louis, MO, USA); sodium carbonate, Na_2_CO_3_ (Sigma-Aldrich, Saint Louis, MO, USA); aluminum chloride; acetic acid (≥99.8% purity, Sigma-Aldrich, Steinheim, Germany); 2,2-diphenyl-1-picrylhydrazyl DPPH (Sigma-Aldrich, Steinheim, Germany). Reference standards: *p*-coumaric acid, caffeic acid, cinnamic acid (Sigma-Aldrich, Steinheim, Germany), pinobanksin, pinocembrin, salicin, galangin, (Sigma-Aldrich, St. Louis, MO, USA). Laboratory electronic scales (“Kern PBS/PBJ”, Kern, Germany); analytical scales (Scaltec SBC 31, Scaltec Instruments GmbH, Göttingen, Germany); water bath (“Harry Gestigkeit GmbH”, Düsseldorf, Germany); laboratory ointment mixer “Unguator 2100” (Germany); pH-meter 766 with electrode Knick SE 104 N (Knick Elektronische Meßgeräte GmbH & Co, Berlin, Germany); viscometer (Vibro viscometer SV-10 (A&D Company Ltd., Tokyo, Japan); magnetic stirrer with a heating surface (IKAMAG C-MAG HS7, IKA-Werke GmbH & Co. KG, Staufen im Breisgau, Germany); spectrophotometer (Agilent 8453 UV-Vis, Agilent Technologies, Santa Clara, CA, USA).

### 5.2. Liquid Extracts of Balsam Poplar Buds Preparation

Liquid extracts of balsam poplar buds were produced using the maceration method. The plant material was crushed, and 50% ethanol (*v*/*v*) is used as the solvent, with a ratio of 1:2 of plant material to solvent. The crushed poplar buds were soaked in the appropriate amount of solvent and macerated for 7 days at room temperature (21 ± 1 °C). The collected extracts were refrigerated for one day to induce the precipitation of contaminants. The extract was then filtered through a filter paper.

### 5.3. Determination of the Total Phenolic Compounds and Flavonoid Content in Balsam Poplar Buds Extract

The determination of the total phenolic compound content in the extracts was performed following the methodology of Singleton, Orthofer, and Lamuela-Raventos (1999), with certain modifications. In total, 150 μL of the sample (the control sample contains the solvent used in the extraction process) was mixed with 2 mL of reagent in a test tube. The reagent consists of 750 μL of 2 M Folin–Ciocalteu reagent. After 3 min, 600 μL of a 75 g/L concentration Na_2_CO_3_ solution was added to the reaction mixture, which was then incubated for 2 h in the dark. The absorbance of the samples was measured using a spectrophotometer at a wavelength of 760 nm [45]. The total phenolic compound content in the liquid extracts was expressed as mg of gallic acid equivalent per gram of dry plant material (mg GAE/g).

For the assessment of the total flavonoid content in the extracts, 5 mL of poplar buds extract was transferred to a 25 mL volumetric flask and diluted with 96% ethanol. From this obtained solution, 1 mL was measured and again transferred to a 25 mL volumetric flask. To this flask, 10 mL of 96% ethanol, 2 mL of AlCl_3_ solution, and one drop of diluted acetic acid were added. The content in the flask was mixed for 20 min, followed by further dilution with 96% ethanol. The absorbance of the samples is measured using a spectrophotometer at a wavelength of 407 nm [45]. The total flavonoid content is expressed as mg of rutin equivalent per gram of dry plant material (mg RE/g).

### 5.4. HPLC Analysis

An examination of the phenolic compounds in the extracts was carried out using high-performance liquid chromatography (HPLC). The chromatographic analysis was performed using a “Waters 2695” system with a diode matrix detector “Waters 996”. The chromatographic column—ACE 5C18 (250 × 4.6 mm). The data obtained were processed using Empower 2 Chromatography Data Software. For the HPLC elution, a mixture of acetonitrile and trifluoroacetic acid was used. The column temperature was maintained at 25 °C, and the flow time was 81 min. An injection volume of 10 µL and a mobile phase flow rate of 1 mL/min were applied. The identification of phenolic compounds was accomplished by comparing their retention times with those of reference materials. Additionally, UV absorption spectra were collected within the wavelength range of 250 to 400 nm. Reference compounds: salicin (R^2^ = 0.9999), *p*-coumaric acid (R^2^ = 0.9999), cinnamic acid (R^2^ = 0.9999), caffeic acid (R^2^ = 0.9999), pinocembrin (R^2^ = 0.9998), pinobanksin (R^2^ = 0.9999), apigenin (R^2^ = 0.9998), galangin (R^2^ = 0.9999) [8].

### 5.5. Modeling of Semi-Solid Pharmaceutical Forms

#### 5.5.1. Gels with Balsam Poplar Buds Extract

Gels of different concentrations (0.5%, 1%, and 2%) are prepared using carbomer as the base. Carbomer powder was dispersed in purified water and mixed using a magnetic stirrer until a homogeneous mass was formed. To neutralize the carbomer, a 10% sodium hydroxide (*v*/*v*) solution was used, and it was added dropwise until the pH reached approximately 6. The sample was continuously stirred until a uniform and transparent gel was obtained. Gels based on carbomer with balsam poplar buds extract were produced at 20%. The first step involved dispersing carbomer powder in purified water and stirring until a uniform mass was formed. Then, the 50% ethanol-based balsam poplar buds extract was added to the prepared base, and the mixture was stirred to ensure even distribution of the active ingredient. To achieve the desired gel consistency, a few drops of 10% sodium hydroxide (*v*/*v*) were added and stirred until a homogeneous mass was obtained.

#### 5.5.2. Emulsions with Balsam Poplar Buds Extract

Sunflower oil was chosen as the oil phase for the emulsion, along with the emulsifier Span 60. Distilled water and the emulsifier Tween 60 were used for the aqueous phase. Appropriate amounts of sunflower oil, Span 60, and Tween 60 were weighed and measured distilled water added. Span 60 was dissolved in sunflower oil, while Tween 60 was dissolved in water. Both phases were alternately heated in a water bath. Once homogeneous masses were formed, the balsam poplar buds extract was gradually added to the oil phase and mixed until a uniform mass was achieved. The oil and water phases were combined and mixed to obtain the emulsion. The resulting emulsion contained 20% liquid balsam poplar buds extract.

#### 5.5.3. Emulgels with Balsam Poplar Buds Extract

Emulgels were prepared using carbomer gels as the gelled aqueous phase. First, an emulsion was produced. Appropriate amounts of sunflower oil, Span 60, Tween 60, and distilled water were weighed and measured. Span 60 was dissolved in sunflower oil, while Tween 60 was dissolved in distilled water. Both mixtures were heated in a water bath. Once homogeneous masses were formed, the balsam poplar buds extract was gradually added to the oil phase and mixed until a uniform mass was achieved. The oil and water phases were combined and mixed to obtain an emulsion. The gelled phase was then added to the prepared emulsion. Mixing continued until a homogenous consistency was achieved. Emulgels were produced, containing 20% liquid balsam poplar buds extract. All experimental formulations were stored in a refrigerator (5 ± 1 °C).

### 5.6. Assessment of Physicochemical Parameters of Semi-Solid Pharmaceutical Formulations

The pH value of the prepared semi-solid pharmaceutical formulations (gels, emulsions, emulgels) was determined using a pH meter. A certain amount of the formulation was placed in a container, and an electrode inserted to measure the pH value. The pH measurement of each semi-solid pharmaceutical form was repeated three times. The viscosity of the semi-solid pharmaceutical formulations is measured using a viscometer. The semi-solid preparation at room temperature was placed into the viscometer cell. A sensory plate was lowered into the cell to measure the formulation’s viscosity.

### 5.7. In Vitro Release Test of Phenolic Compounds from Semi-Solid Pharmaceutical Formulations

The release test of phenolic compounds from gels, emulsions, and emulgels was conducted using Franz-type modified diffusion cells. Prior to the experiment, the semi-permeable membrane was pre-soaked in purified water for 12 h. Two solutions were employed for the release test—a donor solution and an acceptor solution. The donor solution consisted of 1.0 g of the semi-solid preparation and was placed in the diffusion cell. The acceptor solution comprised purified water and 30% ethanol (*v*/*v*). The experiment was conducted at a temperature of 37 ± 0.5 °C. For assessing the quantity of released phenolic compounds, 1.5 mL of samples was taken from the acceptor solution at intervals after 1, 2, 3, 4, and 6 h. Subsequently, the amount of released phenolic compounds was determined using a spectrophotometric method based on the previously described methodology.

### 5.8. Assessment of the Antioxidant Activity of Semi-Solid Pharmaceutical Formulations

The antioxidant activity of semi-solid pharmaceutical formulations was assessed using the DPPH (2,2-diphenyl-1-picrylhydrazyl) radical scavenging assay. In order to evaluate the antioxidant activity of semi-solid formulations, the method was applied to the collected fraction after a 6 h of an in vitro release test. A weight of 0.0012 g of DPPH was accurately measured and dissolved in 50 mL of 96% ethanol. The reaction mixture was protected from light, and the solution allowed to fully dissolve the DPPH radical. The absorbance of the sample was measured using an Agilent 8453 UV-Vis spectrophotometer at a wavelength of 517 nm. The reaction mixture diluted until the absorbance reached 0.8. As a reference solution, 96% ethanol was used. 10 µL of the sample (the fraction derived from the tested semi-solid formulations following an 8 h in vitro release test) was introduced into 3 mL of the DPPH solution. The prepared samples were kept in the dark for 15 min, shaken, and then further incubated for an additional 15 min. The absorbance was measured using a spectrophotometer at a wavelength of 517 nm [46].

The percentage of antiradical activity was determined using the following formula [47]:

Scavenging effect (%) = ((A − B)/A) × 100

A represents the absorption of the DPPH radical without the test sample; B represents the absorbance of the DPPH radical with the test sample.

### 5.9. Antimicrobial Activity

Antimicrobial testing was conducted under sterile conditions. The antimicrobial efficacy of the tested samples was assessed by the well-diffusion method. The resistance of natural material-based preparations was evaluated on Mueller–Hinton agar (Mueller-Hinton Agar II, BBL, Cockeysville, MD, USA) with established strains of *Pseudomonas aeruginosa* ATCC 27859, *Staphylococcus aureus* ATCC 25923, *Escherichia coli* ATCC 29522, *Enterococcus faecalis* ATCC 29212, and *Candida albicans* ATCC 60193. Sterile Petri dishes with a diameter of 85 mm were filled with 20 mL of agar each. Following agar solidification, the wells were filled with the tested samples (100 µL). The cultures were then incubated at 37 °C in a thermostat for 24 h, and subsequently, microbial growth throughout the entire agar volume was assessed. A 0.5% chlorhexidine gel was used as a positive control.

### 5.10. Statistical Analysis

The research data were analyzed using Microsoft Office Excel 2013, SPSS 25.0, and SigmaPlot 13.0. Mean values and standard deviations were reported based on three consecutive tests. To determine statistically significant differences between the compared data, a one-way ANOVA was applied. If the variances of independent variables were found to be equal, Tukey’s multiple comparison test was used. Statistically significant differences were considered at *p* < 0.05.

## Figures and Tables

**Table 1:** Composition of the gels, emulsions, and emulgels with balsam poplar buds (quantity in formulation g/100 g).

	Quantity in Gel g/100 g		
Exipients	G1	G2	G3
Balsam poplar buds extract	20	20	20
Carbomer 980	0.5	1	2
10% NaOH solution	3–4 drops	3–4 drops	3–4 drops
Purified water	ad 100	ad 100	ad 100
	**Composition of emulgels, quantity in emulgel g/100 g**		
	Eg1	Eg2	Eg3
Balsam poplar buds extract	20	20	20
Span 60	1.75	1.75	1.75
Sunflower oil	18.18	18.18	18.18
Tween 60	1.89	1.89	1.89
Purified water	ad 60	ad 60	ad 60
Carbomer 980	0.5	1	2
10% NaOH solution	3–4 drops	3–4 drops	3–4 drops
Purified water	ad 40	ad 40	ad 40
	**Composition of emulsions, quantity in emulsions g/100 g**		
	E1		
Balsam poplar buds extract	20		
Span 60	4		
Sunflower oil	45		
Tween 60	5		
Purified water	ad 100		

Bases of semi-solid formulations of the same composition, without active ingredient, were produced, respectively, by replacing the active substance with an appropriate amount of purified water. Formulations without an active substance are coded accordingly by inserting a 0 symbol in front of the experimental formulation coding (0G1, 0G2, 0G3, 0Eg1, 0Eg2, 0Eg3, 0E1).

**Table 2:** The physicochemical properties of experimental empty formulations and formulations with balsam poplar buds liquid extract (*n* = 3, mean, SD).

	0 G1	0 G2	0 G3	0 Eg1	0 Eg2	0 Eg3	0 E1
pH	5.76	5.98	5.91	5.17	5.54	5.28	4.61
SD	0.06	0.04	0.05	0.04	0.06	0.05	0.04
Viscosity (mPa·s)	248.5	255.2	458.1	194.7	1264.3	2481	2666.1
SD	13.3	14	38.3	9.4	85	196.6	188
	**G1**	**G2**	**G3**	**Eg1**	**Eg2**	**Eg3**	**E1**
pH	5.93	5.93	5.95	5.13	5.66	5.3	4.65
SD	0.06	0.06	0.07	0.05	0.15	0.1	0.06
Viscosity (mPa·s)	237.5	237.5	408.4	202.8	1271.6	2322.4	2641
SD	9.7	9.7	14.3	5.2	41.3	92.7	96.3
Appearance	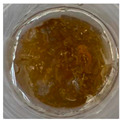	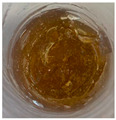	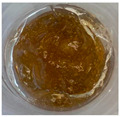	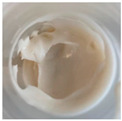	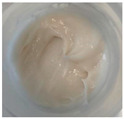	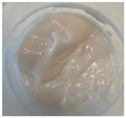	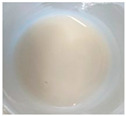

**Table 3:** Antimicrobial activity of *p*-coumaric acid, balsam poplar buds extract, and semi-solid formulations (*n* = 3, mean, SD).

	*Escherichia coli* ATCC 29522 ⊘ mm	*Pseudomonas aeruginosa* ATCC 27859 ⊘ mm	*Staphylococcus aureus* ATCC 25923 ⊘ mm	*Enterococcus faecalis* ATCC 29212 ⊘ mm	*Candida albicans* ATCC 6193 ⊘ mm
*p*-coumaric acid (500 µg/mL)	6.5 ± 0.5	NI	11.8 ± 0.8	10.7 ± 0.7	11.2 ± 0.3
Balsam poplar buds extract	7.8 ± 0.3	NI	20.8 ± 1.0	16.2 ± 1.0	14.8 ± 0.3
G1	NI	NI	5.8 ± 0.3	5.0 ± 0.5	<5
G2	NI	NI	5.7 ± 0.3	<5	<5
G3	NI	NI	5.5 ± 0.5	<5	<5
Eg1	NI	NI	5.8 ± 0.3	<5	<5
Eg2	NI	NI	5.6 ± 0.7	<5	<5
Eg3	NI	NI	5.3 ± 0.6	<5	<5
E1	NI	NI	<5	<5	NI
0.5% chlorhexidine gel	19.3 ± 0.6	16.0 ± 0.9	22.3 ± 0.6	20.2 ± 1.3	12.5 ± 0.5

## Data Availability

All data are available in a publicly accessible repository.
